# A Pilot Investigation of the Social Attention and Communication Surveillance (SACS) Tool for the Early Identification of Autism in Tianjin, China (SACS-C)

**DOI:** 10.3389/fneur.2020.597790

**Published:** 2020-11-16

**Authors:** Josephine Barbaro, Chongying Wang, Jing Wang, Gongshu Liu, Ying Liang, Ji Wang, Ifrah Abdullahi, Cheryl Dissanayake

**Affiliations:** ^1^Olga Tennison Autism Research Centre, School of Psychology and Public Health, La Trobe University, Melbourne, VIC, Australia; ^2^Department of Social Psychology, Zhou Enlai School of Government, Nankai University, Tianjin, China; ^3^Tianjin Women and Children's Health Centre, Tianjin, China; ^4^Yangzhou Maternal and Child Health Hospital, Tianjin, China; ^5^Harbin Children's Hospital, Harbin, China

**Keywords:** early detection, screening, autism spectrum disorder, developmental surveillance tool, China

## Abstract

**Introduction:** Autism spectrum disorder (ASD) comprises difficulties in social communication and restrictive and repetitive behaviors. Despite an increased global prevalence, little remains known about early detection and diagnosis of autism in Mainland China. Our aim was to conduct a pilot investigation of the implementation of an Australian tool, Social Attention and Communication Surveillance (SACS), in Tianjin, China (SACS-C) by trained professionals to identify autism early compared to the Checklist for Autism in Toddlers-23 (CHAT-23) completed by parents and professionals.

**Materials and Methods:** A total of 10,514 children were monitored across 61 Community Health Service Centres in six Tianjin districts on the SACS-C at 12, 18, and 24 months of age following a half-day training of 225 child health practitioners. Children deemed at “high likelihood” for autism on either the SACS, CHAT-23, or both, were referred for developmental assessments at the Tianjin Women and Children's Health Centre (TWCHC).

**Results:** A total of 87 children (0.8%) were identified at “high likelihood” on the SACS-C, of whom 57 (66%) were assessed for autism; 24 children were subsequently diagnosed with autism (42.1%), and the remaining 33 (57.9%) were diagnosed with developmental and/or language delays. The SACS-C had a positive predictive value (PPV) of 42.1%, a negative predictive value (NPV) of 99.8%, and sensitivity and specificity of 53.3 and 99.7%, respectively. Only 21 children were identified at “high risk” for autism on the CHAT-23 (0.2%), over four times fewer children than the SACS-C, with 14 children assessed for autism (66%); nine were diagnosed with autism (64.3%) and the remaining five children were diagnosed with developmental and/or language delays. The CHAT-23 had an overall PPV of 64.3%, NPV of 99.6%, sensitivity of 27.3%, and specificity of 99.9%.

**Conclusion:** This was the first large-scale study identifying autism in 12–24-month-old children in China. We ascertained the feasibility of training community health practitioners to monitor infants and toddlers for the early signs of autism, and determined the effectiveness of their use of SACS-C which had a better balance between accuracy and sensitivity in detecting autism in contrast to the CHAT-23 which missed the majority of children with autism (72.7%) vs. the SACS-C (46.7%). Given the emphasis on identifying as many children with autism as possible in Mainland China, SACS-C was identified as the tool of choice by the TWCHC. However, more work is needed to improve the psychometric properties in using the SACS-C in Mainland China so that it is comparable to its use in Australia.

## Introduction

Autism spectrum disorder (ASD) comprises significant difficulties in social attention, communication and the presence of sensory and restrictive and repetitive behaviors (1). Early developmental surveillance and screening plays a vital role in the early identification, detection and diagnosis of autism, which allows access to early intervention, leading to better child outcomes and improved quality of life (2–5). In Mainland China, early clinical manifestations and symptoms of autism are not widely recognized, often being described as the “lonely disease” (6). In 1982, Dr. Tao Guotai from Nanjing Brain Hospital reported the first four cases of autism in Mainland China (6). Increasing numbers of children are now diagnosed with autism in China, particularly following the improved knowledge and awareness about this condition (7).

The prevalence of autism in the US was recently reported to be 1 in 54 children aged 8 years (8), whilst in the UK it is 1 in 64 (9), Australia 1 in 70 (10), and 1 in 38 in South Korea (11). There remains limited knowledge about the prevalence of autism in Mainland China. A meta-analysis of 18 studies found a wide range in prevalence rates from 2.8 to 30.4 per 10,000, with the pooled prevalence of autism being 12.8 per 10,000 (95% CI: 9.4–17.5) (12), much less than that reported above. More recently a meta-analysis in 2018 found a pooled ASD prevalence of 39.2 per 10,000 (95% CIL 28.4–50.0) and specific prevalence of autism as 10.2 per 10,000 (95% CI: 8.5–11.9) (13). Furthermore, a 2019 study used the Childhood Autism Spectrum Test (CAST) screening tool to ascertain autism prevalence in the Chinese cities of Jilian City, Shenzhen City, and Jiamusi City, finding that autism prevalence estimates were similar to Western prevalence rates in Jilian City (1.08%; 108 per 10,000) but lower in Shenzhen City and Jiamusi City with rates of 0.42% (42 per 10,000) and 0.19% (19 per 10,000), respectively (14).

Sun et al. (15) found the strongest determinant of the variability in prevalence estimates was the screening tool used, and found that studies using the Autism Behavior Checklist (ABC) (16), and the Clancy Autism Behavior Scale (CABS) (17), produced lower prevalence estimates, whilst studies that used the Checklist for Autism in Toddlers (CHAT) (18) reported higher prevalence estimates for autism (15). The authors also noted age at screening as another strong determinant in the prevalence estimates. Fifteen of the 18 studies focused on children screened between the ages of 2–6 years and a further seven focused on children aged 6–14 years and older. Whilst most of the studies included in the systematic review were young children, mean age at diagnosis for children in Mainland China was not reported (15). However, a recent study did report the mean age at diagnosis in Mainland China, with an average age at diagnosis being 3.3 years for Chinese children aged 6–14 years of age (19).

The CHAT (18) and its modified versions (M-CHAT, CHAT-23) are frequently used screening tools in Chinese populations in Mainland China (15, 20). The CHAT is more rigid with a specific applicable age of 18-months; it is a nine-item questionnaire for parents and contains five child observations by professional (18). It has since been further validated, evaluated and modified into the M-CHAT (21), and CHAT-23 versions, with the latter designed for Chinese children (22). CHAT-23 has a validity and reliability scores of 94 and 88%, respectively (23), as well as a sensitivity and specificity of 93 and 85% (22). Despite these seemingly high sensitivities and specificities reported, the age groups screened were wide (or unclear), and none of these studies were exclusively conducted in low-risk, community-based populations. There is, therefore, a gap in the literature on developmental surveillance and community-level screening procedures for infants and toddlers in the general population in China. There is also a lack of professional education available to Chinese primary-care professionals on the early signs of autism (24).

A recent systematic review jointly undertaken by Australian and Chinese scholars (24) reported that whilst screening tools currently used in China have reasonable psychometric properties for identifying autism in clinical populations, there appear to be no studies undertaken with community-based samples. They stressed the need to align the screening and diagnostic systems in Mainland China with best practice in autism screening and diagnosis (25–29). Prioritizing the need for community-based screening in the general population with psychometrically and culturally validated tools is needed together with follow-up of children deemed at high-likelihood of autism at the community level so that they are assessed and diagnosed by a specialized multidisciplinary clinical team (24).

A developmental surveillance tool designed for use in low-risk populations within community-based settings is the Social Attention and Communication Surveillance (SACS) tool. Designed and implemented in Australia, this tool has an excellent Positive Predictive Value (PPV; 81–83%), Negative Predictive Value (99%), Sensitivity (82–84%), and Specificity (99–99.5%) for identifying children with autism between 11 and 30 months of age (26, 30). Moreover, following diagnosis at age 24 months using gold standard tools, diagnostic stability is high at 88.3% between 24 and 48 months of age (25). On the strength of these findings, the SACS tool has been implemented state-wide throughout the universal Victorian and Tasmanian Maternal and Child Health (MCH) Services in Australia, where children are routinely monitored 10 times from birth to 3.5 years. The SACS is administered at 12, 18, and 24 months of age by trained MCH Nurses to identify the early signs of autism during children's routine check-up (30). Importantly, the availability of universal developmental surveillance of babies by medical professionals in China, undertaken within Women and Child Health Centres, provides an ideal platform for monitoring the early signs of autism to promote early identification and diagnosis of autism.

Tianjin is the fourth largest city in China, consisting of 16 county-level administrative areas. Over 100,000 babies are born in Tianjin every year, which, based on the estimated prevalence of autism was 27.5 per 10,000 (31), equates to 1,000–2,000 babies potentially born with autism each year. A thoroughly developed women and child health care system in this city, the Tianjin Women and Children's Health Centre (TWCHC), has made it an ideal test site for the implementation of screening for various child conditions such as congenital heart disease (32), developmental dysplasia of the hip (33), and congenital cataract (34). However, developmental surveillance and screening for autism had not yet been implemented.

Our study objective was to conduct a pilot investigation of a Mandarin translation of the SACS, –SACS-China (SACS-C) – in Tianjin, by comparing the outcomes of implementing the SACS-C with the CHAT-23, as described above, and which has been widely used with Chinese children (22, 35, 36). The study comprised two aims: firstly, to ascertain the feasibility of training early child health experts to monitor babies and toddlers for early signs of autism in Tianjin; and, secondly, to determine the performance of two tools (SACS-C and CHAT-23) to enable selection for use in early identification for autism in the TWCHC.

## Method

### Study Setting

Tianjin has a three-level maternal and child health care system, consisting of a city level administrative centre (the TWCHC), Women and Children's Health Centres at a district level, and the community level health centres (CHC). In Tianjin, children's health status and development are monitored in the community health centers by qualified medical health practitioners. The CHC services are offered to all families with children younger than 7 years, with an emphasis on child health surveillance and screening (37). As part of this service, routine health checks for children in the community are scheduled from birth to 7 years of age. It is expected that children under 12 months are examined every 3 months, children between 12 and 36 months are examined every 6 months, and children over 36 months are examined once a year (37). Every year, over 90% of babies in Tianjin access the CHC service soon after birth, with attendance remaining relatively high within the first 2 years; this service has enormous potential to identify infants at high-likelihood of autism.

### Participants

**Figure 1:**
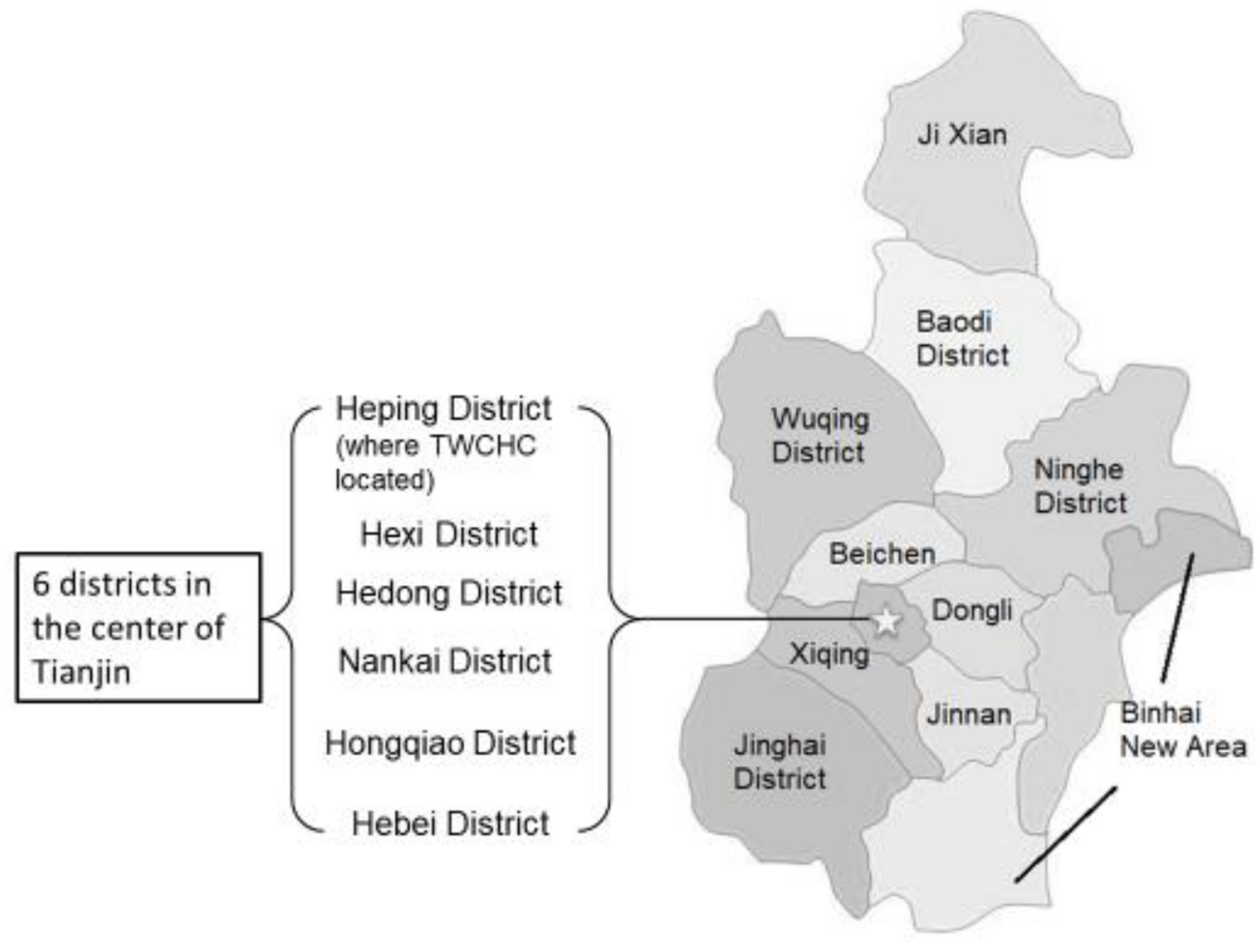
The map of Tianjin, highlighting the six urban districts involved in the current study.

From May 2013 to July 2014, a total of 10,514 children were monitored through 61 CHCs in six selected districts in the urban areas in Tianjin (see star in Figure 1). In 2010, 4.3 million out of 13 million residents lived in the six central urban districts. The districts in this study were chosen based on proximity to facilitate ease of referral to the diagnostic center at the TWCHC, which is in the city center.

While all 10,514 children were monitored with the SACS-C, only a subset (*n* = 6,744; 64%) were also screened with the CHAT-23. Many children in the original SACS studies (26, 30) were seen at each of the 12, 18, and 24 months checks. However, in this pilot study, children were only monitored twice on the SACS-C (at 12, 18 and/or 24 months) due to funding restrictions. Initially children were monitored on the SACS-C at 12-months (*n* = 3,178), 18-months (*n* = 3,757), and 24-months (*n* = 3,579) of age. As the SACS-C is a developmental surveillance tool administered at different time points, the majority of the cohort (66%) initially monitored at 12, 18, or 24 months were also monitored again by the health practitioners 6 months later; 79% of 12-month-olds (*n* = 2,497), 78% of 18-month-olds (*n* = 2,911), and 42% of 24-month-olds (*n* = 1,494). Children within the age limits of the CHAT-23 at 18-months (*n* = 3,683) and 24-months (*n* = 3,061) were only monitored once, given its use as a “once-off” screening tool. The average age of all children monitored in the study was 18.70 months (SD 4.99), with the sample comprising 52% boys and 48% girls. Detailed age, gender and assessment characteristics are shown in Table 1.

**Table 1:** Demographics characteristics of children administered the SACS-C and CHAT 23.

		**SACS-C**			**CHAT-23**		
**Age (months)**	**12**	**18**	**24**	**Overall**	**18**	**24**	**Overall**
*n*	3,178	3,757	3,579	10,514	3,683	3,061	6,744
Age M (SD)	12.36 (0.60)	18.44 (0.70)	24.6 (1.24)	18.70 (4.99)	18.45 (0.69)	24.23 (0.33)	21.08 (2.93)
**Gender**							
Male (%)	1,653 (30.2)	1,954 (35.7)	1,861 (34.1)	5,468 (100) (52.0)	1,915 (54.6)	1,592 (45.4)	3,507 (100) (52.0)
Female (%)	1,525 (30.2)	1,803 (35.7)	1,718 (34.1)	5,046 (100) (48.0)	1,768 (54.6)	1,469 (45.4)	3,237 (100) (48.0)
Total (%)	3,178 (30.2)	3,757 (35.7)	3, 579 (34.1)	10,514 (100)	3,683 (54.6)	3,061 (45.4)	6,744 (100) (100)

*CHAT-23, Checklist for Autism in Toddlers-23; SACS-C, Social Attention and Communication Surveillance-China Tool; n, number of participants; M (SD), mean (standard deviation)*.

### Measures

#### Translation of SACS Checklists

For effective implementation in Tianjin, the SACS was translated from English into Chinese by one of the authors (CW) and further validated by a practitioner from the TWCHC. The SACS-C was then back translated to English, this English version checked by the first author (JB) and this process was repeated twice between CW, Chinese colleagues and JB, with modifications made to be in line with the “meaning” of the original instrument. Authors CW and JB then evaluated both the English and Chinese versions to ensure these were comparable in meaning. A summary of the behaviors monitored with the SACS-C, highlighting the “key items,” are presented in Supplementary Table 1.

#### Training of Community Practitioners on SACS-C

In March 2013, 225 child health practitioners from 61 communities within the six districts in Tianjin received a 3-hour-training workshop by the authors of the SACS (JB & CD). The workshops focused on typical and atypical social-communicative development, the early signs of autism, and the administration of the SACS items. Simultaneous translation from English to Mandarin was undertaken during the workshops (by CW), with all written content also translated and then back translated by the CW and JB.

The SACS authors (JB and CD) also observed administration of the SACS-C with two children at each of the ages of 12, 18, and 24-months, undertaken by a number of the trained health practitioners at TWCHC, and provided in-person feedback on these administrations. These health practitioners then assisted CHC practitioners in any queries relating to SACS-C administration and scoring.

#### SACS-C Implementation

Following training, the SACS-C was implemented as part of the routine health checks in the CHCs. Community health practitioners initially undertook a physical examination of the child, and the child was monitored on the SACS-C in the presence of a parent/caregiver. The practitioners, who had been trained on how each item was to be administrated at each developmental age, recorded whether the child's behaviors were typical or atypical (as opposed to present or absent) on a form provided for each child. Children were considered at “high-likelihood” for autism if they did not engage in three of the five “key” items at 12, 18, and/or 24-months-of-age. Practitioners were instructed to administer items up to a maximum of three times (e.g., calling a child's name). In the minority of cases where practitioners were unable to elicit a behavior because of the child being ill, distressed, or asleep, detailed parental/caregiver report was used to mark the item as typical or atypical. Children who were identified as “high-likelihood” for autism were referred to the TWCHC for a follow-up developmental and diagnostic assessment for autism by two autism specialist pediatricians.

#### CHAT-23 Training and Implementation

The health practitioners were also trained on the use of the CHAT-23 by one of the authors (CW), who is a native Chinese speaker. CHAT-23 is popular in Chinese-speaking areas, and the Chinese version of the test is available. The training focused on how to identify the passing or failing in each item, and the referral standards. The CHAT-23 comprises two parts: Part A is a parent questionnaire with 23 questions regarding children's behaviors, and Part B comprises seven-key item scored based on observations of the child, conducted by the health practitioner. If a child fails six or more items of the total of 23 parent completed items in Part A, they are administered Part B. If the child had two or more failed items in Part B, the child was identified as “high-likelihood” for autism on the CHAT-23 and referred to the TWCHC for a further assessment. Notably, unlike the SACS-C, which is a developmental surveillance tool administered across the second year of life, the CHAT 23 is administered only once between 18 and 24 months of age and is appropriate after 18 months of age, as it is a screening tool designed to be administered at one point in development.

### Procedure

Study recruitment was conducted through advertisements on clinic noticeboards, as well as brochures on the early signs of autism displayed and issued to parents in the visiting room. The SACS-C and CHAT-23 were introduced during children's routine health checks. Firstly, parents filled out Part A of the CHAT-23 in the waiting room (if the child was aged between 18- and 24-months). Children then underwent their routine health checks with the health practitioner, including measurement of height and weight. The health practitioners reviewed parent responses on Part A of the CHAT 23 and followed this with Part B of CHAT-23 (if the child had failed Part A); the SACS-C was then administered by the practitioner for all children. If a child was deemed “high-likelihood” for an autism on either the SACS-C, CHAT-23, or both, the health practitioners advised parents about their concerns regarding the child's development in social attention and communication. Parents were told that the monitored behaviors were important developmental milestones that need to be assessed further, and they were then referred to the TWCHC for a further developmental and diagnostic assessment for autism.

This study was approved by the Tianjin Women and Children's Health Centre (TWCHC) Human Ethics Committee.

### Data Collection and Quality Control

Quality Control was undertaken during the entire data collection process. During the early stages of data collection, nominated staff from TWCHC and students from Nankai University (NU) were sent to the six districts, with one person allocated per district. They assisted the community health practitioners to correctly administer, score, and use of the SACS-C and CHAT-23 with the children. Furthermore, one staff member from TWCHC (JW) and Nankai University (CW) visited approximately 35% of the communities, thus ensuring correct administration of the two tools in all six districts, and accurate completion of the checklists. They also provided feedback to the health practitioners on the use of the tools and the referral procedure. Additionally, mid-way through data collection, a TWCHC staff member (JW) and one student from Nankai University re-visited ~30% of the communities to check project implementation.

The SACS-C and CHAT-23 data sheets were initially stored in secure cabinets in the local CHCs and transferred to TWCHC at the end of the data collection phase, where they were stored in a secure cabinet. Each child was assigned a unique identification number, used to link child and assessment details. Health practitioners also entered the data from the record forms into a database at each CHC. Both the hard copy form and the database from each different district was then collected, and data entered for a second time at the TWCHC. Students from NU were involved in the second data entry process. Epidata 3.1 was used for the double data entry, and all the statistical analysis was undertaken using SPSS 21.0; the final database was stored in an encrypted computer at TWCHC and Nankai University.

### Assessment Protocols for Children at “High-Likelihood” for Autism

The diagnostic procedure for autism in China involves a four-step process: (1) Collecting the medical history, including the clinical symptoms related to autism, the child's growth and developmental information, and family history; (2) Conducting cognitive assessments, including observing the child's behavioral symptoms and communication. Based on their clinical experience, each physician sets up an environment and activities to observe the child's behaviors (no one specific standard applied); (3) physical and neurological examination, including laboratory tests and administration of psychological assessments to assist the diagnosis if needed; (4) Before diagnosing as autism, other conditions such as language developmental disorders, intellectual disability, childhood schizophrenia and mental illnesses and other developmental disorders are excluded (differential diagnosis).

Two pediatricians from the TWCHC [Dr. Liang, Associate Chief Physician has 14-years of experience in diagnosing children's psychological and behavior disorders, and was trained on the Autism Diagnostic Observation Scale (ADOS); Dr. Yao, Chief Physician, has more than 10 years of experience in diagnosing children's psychological and behavior disorders] undertook the assessments and diagnosis of the referred children. The assessment tools commonly used with the referred children included the ASD Behavior Checklist (ABC) (17), Gesell Development Scale (GDS) (38), and Infants-Junior Middle School Students Social-Life Abilities Scale (S-M scale) (39). These tests were not used on all children but selected at the discretion of the clinicians based on signs the children were displaying. A final diagnosis of autism or Non-autism was then made on the basis of the above tests and clinical judgment. The above assessment scales conducted for the children identified “high-likelihood” on SACS-C and CHAT-23 are listed in Table 2.

**Table 2:** Sample characteristics of assessed children grouped according to age and diagnosis at each health check 12, 18, and 24 months.

	**Group**					
	**Autism**		**DD/LD**		**TD**	
**SACS-C at 12-month-check**						
SACS-C (*n* = 16)	3		9		4	
Mean age of identification (SD)	12.09 (0.11)		12.27 (0.24)		12.11 (0.19)	
Mean age of assessment (SD)	15.05 (2.86)		13.07 (1.09)		15.70 (4.53)	
Gender (male: female)	3:0		6:3		2:2	
**Tests**						
ABC	*n* = 1	78.00	*n* = 3	19.33 (8.51)	*n* = 1	18.00
Development scale	*n* = 2	68.65 (10.96)	*n* = 8	78.13 (8.56)	*n* = 4	89.63 (7.36)
S-M	n = 1	9.0	*n* = 4	9.75 (0.50)	*n* =1	10.00
**SACS-C at 18-month-check**						
SACS-C (*n* = 20)	7 (5 “high-likelihood” on CHAT)		10 (2 “high-likelihood” on CHAT)		3 (1 “high-likelihood” on CHAT)	
Mean age of identification (SD)	18.50 (0.56)		18.06 (0.43)		18.23 (0.26)	
Mean age of assessment (SD)	21.38 (7.097)		22.03 (10.54)		22.18 (1.18)	
Gender (male: female)	7:0		8:2		1:2	
**Tests**						
ABC	*n* = 2	47.50 (7.78)	*n* = 6	31.83 (18.23)	*n* = 2	18.50 (7.78)
Development scale	–	–	*n* = 6	78.10 (8.68)	*n* = 2	88.60 (3.68)
S-M	*n* = 5	9.20 (0.48)	*n* = 8	8.75 (3.66)	*n* = 2	10.00 (0.00)
**CHAT-23 at 18-month-check**			
CHAT-23 (*n* = 8)	5 (5 “high-likelihood” on SACS-C)		2 (2 “high-likelihood” on SACS-C)		1 (1 “high-likelihood” on SACS-C)	
Mean age of identification (SD)	18.48 (0.68)		18.12 (0.12)		18.53	
Mean age of assessment (SD)	22.39 (8.43)		34.89 (23.60)		23.03	
Gender (male: female)	5:0		2:0		1:0	
**Tests**			
ABC	*n* = 2	47.50 (7.78)	*n* = 1	58.00	*n* = 1	24.00
Development scale	–	–	*n* = 1	77.90	*n* = 1	91.20
S-M	*n* = 3	9.33 (0.58)	*n* = 1	12.00	*n* = 1	10.00
**SACS-C at 24-month-check**			
SACS-C (*n* = 21)	14 (4 “high-likelihood” on CHAT)		6 (0 “high-likelihood” on CHAT)		1 (0 “high-likelihood” on CHAT)	
Mean age of identification (SD)	24.98 (1.92)		27.09 (2.78)		26.74	
Mean age of assessment (SD)	25.82 (2.13)		27.92 (2.16)		27.04	
Gender (male: female)	13:1		3:3		1:0	
**Tests**			
ABC	*n* = 12	44.08 (17.93)	*n* = 1	54.00		
Development scale			*n* = 4	65.65 (18.03)	*n* = 1	91.10
S-M	*n* = 9	7.22 (0.44)	*n* = 2	8.00 (1.41)		
**CHAT-23 at 24-month-check**			
CHAT-23 (*n* = 6)	4 (4 “high-likelihood” on SACS-C)		2 (0 “high-likelihood” on SACS-C)		0	
Mean age of identification (SD)	24.22 (0.11)	24.38 (0.33)	–
Mean age of assessment (SD)	25.06 (1.65)	34.27 ± 14.22	–
Gender (male: female)	3:1	0:2	–
**Tests**			
ABC	*n* = 4	56.25 (11.76)	*n* = 1	51.00	–	
S-M	*n* = 1	7.00	–	–	–	

ABC, ASD Behavior Checklist; CHAT-23, Checklist for Autism in Toddlers-23; Development scale, Gesell Development Scale; S-M, Infants-Junior Middle School Students Social-Life Abilities Scale; SACS-C, Social Attention and Communication Surveillance-China tool; n, number of participants; SD, standard deviation. **Tests, These tests were not used on all children but selected at the discretion of the clinicians based on signs the children were displaying. –, not applicable/administered.

### Follow-Up

Approximately 80% of the children monitored by the health practitioners in this study were followed-up in kindergartens from the six districts in Tianjin when they were aged between 3 and 4 years of age, to identify any “false negatives” from the surveillance and screening procedure undertaken between 12- and 24-months of age. JW from TWCHC and at least two trained practitioners from every district-level Women and Children's Health Centers visited the municipal and district kindergartens, respectively. Firstly, observation sheets were issued to the teachers in advance, which listed eight atypical behaviors and/or developmental concerns (see Supplementary Table 2), and they were asked to nominate children demonstrating those behaviors. Secondly, interviews were conducted with teachers regarding children who were identified as showing atypical behaviors to obtain more information about their behavior and development. The interviewers then observed the nominated children in the class, focusing on their social-communication skills and overall development. If the children were indeed showing atypical behaviors/development, they were referred to TWCHC for a further assessment and diagnosis by the two pediatricians.

### Early Intervention

Children diagnosed with autism were referred to an autism intervention organization. Children who were diagnosed with other delays and disorders were referred to one of the child development intervention institutes, and their parents were taught some simple interventions by the clinicians, such as increasing social activities with other peers, encouraging more eye contact, and applying effective reinforcers to decrease behavioral problems.

## Results

### Children Tested on Both the SACS-C and CHAT-23

Of the children assessed on both the SACS-C and CHAT-23 (*n* = 6,744), 21 were flagged as “high-likelihood” on the CHAT-23, and 52 were flagged as “high-likelihood” on SACS-C, with 17 children identified as being at “high-likelihood” on both tools (see Table 4). The Positive Predictive Value (PPV) of children with “high-likelihood” on both SACS-C and CHAT-23 was 81.0%, whilst the Negative Predictive Value (NPV) was 99.2%.

### Psychometric Properties of SACS-C

Of the 10,514 children monitored with the SACS-C, 87 children were identified as “high-likelihood” (0.83% of the sample). Of these children at high-likelihood, 27.6% were identified at 12-months of age, 34.5% at 18-months of age, and 37.9% at 24-months of age. Only 57 (65.5%) of the 87 high-likelihood children were assessed for autism, as 30 families declined the invitation for a developmental assessment (Table 3). Of the 57 children assessed, 24 were diagnosed with autism (42.1%), and 25 (43.9%) children were diagnosed with developmental and/or language delays/disorders (DD/LD); a further 8 (14.0%) children were determined to be typically developing (TD).

**Table 3:** Assessment characteristics of children administered the SACS-C and CHAT 23.

		**SACS-C**			**CHAT-23**		
**Age (months)**	**12**	**18**	**24**	**Overall**	**18**	**24**	**Overall**
*n*	3,178	3,757	3,579	10,514	3,683	3,061	6,744
Assessed (%)	16 (28.1)	20 (35.1)	21 (36.8)	57 (100)	8 (57.1)	6 (42.9)	14 (100)
Autism (%)	3 (12.5)	7 (29.2)	14 (58.3)	24 (100)	5 (55.6)	4 (44.4)	9 (100)
DD/LD (%)	9 (36.0)	10 (40.0)	6 (24.0)	25 (100)	2 (50.0)	2 (50.0)	4 (100)
TD (%)	4 (50.0)	3 (37.5)	1 (12.5)	8 (100)	1 (100)	0	1 (100)
Declined assessment (%)	8 (26.7)	10 (33.3)	12 (40.0)	30 (100)	4 (57.1)	3 (42.9)	7 (100)
Total “high-likelihood” (%)	24 (27.6)	30 (34.5)	33 (37.9)	87 (100)	12 (57.1)	9 (42.9)	21 (100)
PPV Autism %	18.75	35.0	66.7	42.1	62.5	66.7	64.3
PPV all disorders %	75.0	85.0	95.2	86.0	87.5	100.0	92.9

DD/LD, Developmental Delay or Language Delay; TD, Typically Developing (TD); CHAT-23, Checklist for Autism in Toddlers-23; SACS-C, Social Attention and Communication Surveillance-China tool; PPV, Positive Predictive Value; n, number of participants. **Total “high-likelihood” equals to total children deemed high-likelihood for autism.

**Table 4:** The number of children deemed at “high” (positive) and “low” (negative) likelihood for autism following screening on SACS-C and CHAT-23.

	**CHAT positive**	**CHAT negative**	**Total**
SACS-C positive	17	35	52
SACS-C negative	4	6,688	6,692
Total	21	6,723	6,744

*CHAT-23, Checklist for Autism in Toddlers-23; SACS-C, Social Attention and Communication Surveillance-China tool*.

The positive predictive value (PPV) for the SACS-C was 42.1% for autism and 86.0% for all developmental delays/disorders when used between 12 and 24-months of age. At the 2-year post-assessment follow-up, an additional 21 children were identified and diagnosed with autism; these children had previously been identified as “not high-likelihood” on the SACS-C when seen between 12- and 24 months, resulting in a Negative Predictive Value (NPV) of 99.8% for autism. Sensitivity and specificity for autism on the SACS-C was 53.3 and 99.7%, respectively. The estimated prevalence of autism among the study population monitored by the SACS-C (including follow-up) was 0.55%.

### Psychometric Properties of CHAT-23

Of the 6,744 children also monitored with the CHAT-23, 21 children were identified as “high-likelihood” (0.31% of the sample), with 57% identified at 18-months and 43% at 24-months. However, as seven families declined an offer for a developmental assessment, only 14 children at “high-likelihood” for autism was assessed at the TWCHC. Of these, nine were diagnosed with autism (64.3%), four were diagnosed with developmental and/or language delays/disorders (DD/LD), with one child identified as typically developing (TD) (see Table 3). The CHAT-23 had an overall PPV of 64.3% for autism and 92.9% for all developmental delays/disorders. At the 2-year post-assessment follow-up, similar to SACS-C, an additional 24 children were identified and diagnosed with autism among children originally defined as “low-likelihood” on the CHAT-23, thus resulting in an NPV of 99.6%. Sensitivity and specificity for autism on the CHAT-23 was 27.3 and 99.9%, respectively. The estimated prevalence of autism among the study population using the CHAT-23 (including follow-up) was 0.56%.

## Discussion

This is the first large-scale study on developmental surveillance for autism in infants and toddlers among children in China. The findings demonstrated the feasibility of implementing developmental surveillance for autism within the Tianjin, Mainland China. They also indicated that the SACS-C tool was effective in identifying autism in a community-based sample at an early age. The SACS-C was found to have higher sensitivity compared with CHAT-23 (53.33 vs. 27.27%, respectively), but a lower PPV (42.11 vs. 64.29%). For both measures, the PPV increased with increasing age of screening, from 12 to 24 months of age, and at the age of 24 months, the PPV of SACS-C and CHAT-23 were the same (both PPV = 66.7%). A possible explanation is that for older children, the atypical behaviors are more prevalent and detectable by both parents and health practitioners. The specificity and NPV of the two tools were also very similar (SACS 99.7, 99.8%; CHAT-23 99.9, 99.6%, respectively). However, the results showed that the SACS-C identified many more children with autism than the CHAT-23 (0.83 vs. 0.31%), with the latter missing more children during these early years.

Previous studies and meta-analyses have reported considerable variability in prevalence estimates, ranging from 1.8 to 426.4 per 10,000 (12, 15, 40). These studies indicated that compared with estimates of around 1% in developed countries, the reported prevalence of autism in Mainland China is much lower (12, 15, 40). Sun et al. reported an estimated prevalence of 119 per 10,000 among 737 school-age (6–10 years) children (7). In our study population based in Tianjin City, the rate of autism was estimated to be 0.43% (1 in 233) based on the SACS-C and 0.49% on the CHAT-23 (1 in 204). This estimate is similar to the prevalence in Shenzhen City, with an estimate of 0.42% (42 per 10,000 95% CI 20–89) (14). Our lower estimated prevalence rates could possibly be explained by the lack of knowledge of and experience with the early signs of autism, leading to a lower detection rate (24).

When the two screening tools were compared in this study, the SACS-C demonstrated a better balance between accuracy (PPV) and sensitivity in identifying autism in infants and toddlers compared to the CHAT-23. There are also a number of advantages of using SACS-C; firstly, the SACS-C is potentially more objective because the community health practitioners directly observed and rated the SACS-C items, whereby their administration of the CHAT-23 is based on parents responses in the first instance, who are likely to be less knowledgeable about autism. (15) Secondly, the SACS-C had a higher sensitivity, detecting more autism cases in the community-based population, which is essential as it is the ultimate aim of screening (26). Although SACS-C had a lower PPV than the CHAT-23, the higher PPV of the CHAT-23 came at the cost of fewer referrals, and lower sensitivity. Also, when looking at the 24-month data, the SACS-C and CHAT-23 had identical PPVs. Finally, the SACS-C is a developmental surveillance tool, so that repeated monitoring is conducted across the second year of life, ensuring the tool is able to identify children with autism at subsequent checks if they are not initially identified, rather than being a single screen at a given period.

A significant strength of this study was the successful training of community health professionals that enabled the community-based surveillance of infants and toddlers in Tianjin, Mainland China for autism and related conditions. However, there are a few study limitations that should be noted. The lower sensitivity and PPV of SACS-C, compared to the original SACS (30), could be due to a few factors, such as possible cultural differences in administration of the SACS-C, limited knowledge and experience of community health professionals in early autism symptoms presentation and detection prior to this pilot study, differences between the two community health systems, and differences in the diagnostic procedures.

The diagnostic procedures for autism in China differed to those undertaken in Australia and varied according to the pediatricians preference. For example, the diagnostic assessments were not conducted using gold standard diagnostic tools such as ADOS (41), and Autism Diagnostic Interview-Revised (ADI-R) (42). Given that the percentage of children identified as “high-likelihood” on SACS-C (0.83%) was similar to the rate of children at “high-likelihood” for autism in the original SACS (1.04%) (30), it is possible that the diagnostic assessments conducted in Tianjin were not identifying as many children with autism that did indeed have autism, and instead diagnosed children with other conditions instead.

This pilot study implemented the SACS-C in Tianjin, China, and effectively compared its performance with that of CHAT-23 in a large community-based sample. In so doing, the feasibility of successfully training community health practitioners to monitor infants and toddlers for the early signs of autism using SACS-C was established. The SACS-C was found to be efficacious and culturally valid for use with Tianjin infants and toddlers aged 12- to−24-months. The SACS-C revealed a good balance between accuracy and sensitivity in detecting autism compared to the CHAT-23, which missed the majority of children on the spectrum (72.7 vs. 46.7%). Given these findings, it was found that newly-trained community health practitioners can identify and refer more infants and toddlers with the early signs of autism on SACS-C than CHAT-23, indicating that the SACS-C is a viable alternative to be implemented in the CHC system in Tianjin. Based on the findings from this study, the team at the TWCHC selected SACS-C as the preferred autism developmental surveillance tool, such that it was incorporated into the 7-year Tianjin Women and Child Health Plan (2013–2020). Infants and toddlers in Tianjin have since been monitored for autism using the SACS-C following the training of all early child health professionals in Tianjin. However, future research is needed to improve the psychometric properties of the SACS-C in Mainland China so that it is comparable to its use in Australia.

## Data Availability Statement

All datasets generated for this study are included in the article/Supplementary Material.

## Ethics Statement

The studies involving human participants were reviewed and approved by Tianjin Women and Children's Health Centre (TWCHC) Human Ethics Committees. Written informed consent to participate in this study was provided by the participants' legal guardian/next of kin.

## Author Contributions

JiW conducted the analyses, contributed to the interpretation of the results, and drafted the initial manuscript. JB, CW, GL, and CD developed the study design, contributed to data analysis and interpretation, and reviewed drafts, with JB coordinating these tasks. YL conducted the developmental assessment for children referred for assessment. JinW and IA contributed to the literature review and review of the manuscript. All authors contributed to the article and approved the submitted version.

## Conflict of Interest

The authors declare that the research was conducted in the absence of any commercial or financial relationships that could be construed as a potential conflict of interest.
