# Supplementary material for: A Pilot Investigation of the Social Attention and Communication Surveillance (SACS) Tool for the Early Identification of Autism in Tianjin, China (SACS-C)
Source: Front Neurol. 2020 Nov 16;11:597790. doi: 10.3389/fneur.2020.597790 (PMC7701138; doi:10.3389/fneur.2020.597790)
Supplement: Supplementary file 1 [file Data_Sheet_1.docx]

Supplementary Material

# Supplementary Tables

# Supplementary table 1.

# *Behaviours Monitored in the SACS at Each Age, Including “Key Item” for ASD.*

| Behaviour | 12 months | 18 months | 24 months |
| --- | --- | --- | --- |
| Pointing | √ (Key Item) | √ (Key Item) | √ (Key Item) |
| Eye contact | √ (Key Item) | √ (Key Item) | √ (Key Item) |
| Waving bye-bye | √ (Key Item) | √ (Key Item) | √ (Key Item) |
| Response to name | √ (Key Item) | √ | √ |
| Imitation | √ (Key Item) | √ | √ |
| Social communication (showing |  | √ (Key Item) | √ (Key Item) |
| Pretend play |  | √ (Key Item) | √ (Key Item) |
| Follows point | √ | √ | √ |
| Social Smile | √ | √ | √ |
| Conversational babble | √ |  |  |
| Speak 1-3 words | √ |  |  |
| Understands simple instructions | √ |  |  |
| Attending to sounds | √ |  |  |
| Uses 5-10 words |  | √ |  |
| Understands words |  | √ |  |
| Obeys simple instructions |  | √ |  |
| Points to facial features |  | √ |  |
| Loss of skills |  | √ | √ |
| Use 20-50 words |  |  | √ |
| 2-word utterances |  |  | √ |
| Follows simple commands |  |  | √ |
| Parallel play |  |  | √ |
| Interest in other children |  |  | √ |

# Supplementary table 2.

# *Follow up behaviours/concerns monitored by teachers of kindergarten aged children who participated.*

| 1. Language delay; 2. Fine and gross motor delays or poor motor coordination; 3. Poor eye contact; 4. Unsocial, not playing with other children; 5. Not getting along well with others, likely to fight or grab things; 6. Poor behaviour management, not following the instructions of teachers; 7. Noticeable differences in learning or adaptive skills in comparison to peers; 8. Noticeable differences in any other area of development in comparison to peers. |
| --- |
